# Combining genome size and pollen morphology data to study species relationships in the genus *Daucus* (Apiaceae)

**DOI:** 10.1186/s12870-022-03743-1

**Published:** 2022-08-01

**Authors:** Dariusz Kadluczka, Elwira Sliwinska, Ewa Grzebelus

**Affiliations:** 1 Department of Plant Biology and Biotechnology, Faculty of Biotechnology and Horticulture, University of Agriculture in Krakow, al. Mickiewicza 21, 31-120 Krakow, Poland; 2 Laboratory of Molecular Biology and Cytometry, Faculty of Agriculture and Biotechnology, Bydgoszcz University of Science and Technology, al. Kaliskiego 7, 85-796 Bydgoszcz, Poland

**Keywords:** Crop wild relatives, Flow cytometry, Nuclear DNA content, Palynology, Plant systematics, Plant taxonomy

## Abstract

**Background:**

The genus *Daucus* (Apiaceae) comprises about 40 wild species and the cultivated carrot, a crop of great economic and nutritional importance. The rich genetic diversity of wild *Daucus* species makes them a valuable gene pool for carrot improvement breeding programs. Therefore, it is essential to have good knowledge of the genome structure and relationships among wild *Daucus* species. To broaden such knowledge, in this research, the nuclear DNA content for 14 *Daucus* accessions and four closely related species was estimated by flow cytometry and their pollen morphology was analyzed by light and scanning electron microscopy (SEM).

**Results:**

The flow cytometric analysis showed a 3.2-fold variation in the mean 2C values among *Daucus* taxa, ranging from 0.999 (*D*. *carota* subsp. *sativus*) to 3.228 pg (*D*. *littoralis*). Among the outgroup species, the mean 2C values were 1.775–2.882 pg. The pollen grains of *Daucus* were tricolporate, mainly prolate or perprolate (rarely) in shape, and mainly medium or small (rarely) in size (21.19–40.38 µm), whereas the outgroup species had tricolporate, perprolate-shaped, and medium-sized (26.01–49.86 µm) pollen grains. In the studied taxa, SEM analysis revealed that exine ornamentation was striate, rugulate, perforate, or the ornamentation pattern was mixed. At the time of shedding, all pollen grains were three-celled, as evidenced by DAPI staining. We also found high positive correlations between the length of the polar axis (P) and the length of the equatorial diameter (E) of pollen grains, as well as between P and P/E. However, when comparing cytogenetic information with palynological data, no significant correlations were observed.

**Conclusions:**

This study complements the information on the nuclear DNA content in *Daucus* and provides comprehensive knowledge of the pollen morphology of its taxa. These findings may be important in elucidating the taxonomic relationships among *Daucus* species and can help in the correct identification of gene bank accessions. In a broader view, they could also be meaningful for the interpretation of evolutionary trends in the genus.

## Background

The genus *Daucus* L. is a member of Apiaceae, a large, complex, and cosmopolitan family of approximately 466 genera and 3820 species that are especially diverse in temperate regions of Eurasia and North America [1]. Although the Apiaceae family is well defined morphologically by a wide range of distinctive characteristics, allowing its constituent taxa to be unambiguously assigned to the family, taxonomic divisions within the family have been extensively discussed [2]. The cultivated carrot (*D*. *carota* L. subsp. *sativus* Hoffm.) is economically and nutritionally the most significant member of the genus, providing a major source of vitamin A precursors (α- and β-carotene) in the human diet [3]. Based on a morpho-anatomical study by Sáenz Laín [4], *Daucus* has traditionally comprised 20 species and has been divided into five sections: *Daucus* L., *Anisactis* DC., *Platyspermum* DC., *Chrysodaucus* Thell, and *Meoides* Lange. This classification was further extended by Rubatzky et al. [5], who listed 25 species. Recently, a number of molecular analyses involving plastid genes (*rbcL*, *matK*), plastid introns (*rpl16*, *rps16*, *rpoC1*), ribosomal internal transcribed spacer (ITS) sequences, chloroplast and mitochondrial DNA restriction sites, nuclear orthologs, and nuclear single nucleotide polymorphisms (SNPs) have been performed to clarify the phylogenetic relationships among *Daucus* species and their close relatives in the subfamily Apioideae [6–19]. These studies have resulted in the division of the genus into two subclades: *Daucus* I and *Daucus* II. *Daucus* I comprises *D*. *carota* subsp. *carota* L. (the wild ancestor of the cultivated carrot) with all *D*. *carota* subspecies, as well as several *Daucus* species of Mediterranean origin and some species traditionally placed in other genera, whereas *Daucus* II includes the remaining members of the genus. Following a recent taxonomic revision of *Daucus* by Banasiak et al. [16], the genus has been enlarged to include another 18 species from 9 other genera and now contains about 40 species.

The rich genetic diversity of wild species of *Daucus* could be utilized in carrot breeding programs; hence, it is crucial to better understand the genome structure and relationships among these species. Genome size, defined as the amount of DNA in the holoploid genome of an organism [20], is a fundamental biological characteristic, which can provide insight into the evolutionary background of a species. Knowledge of genome size is important in elucidating the taxonomic relationships among species and tracing evolutionary changes. It may also help resolve conflicting hypotheses concerning the origin of polyploids and serve as additional quality control for species identification in germplasm collections. It can also be useful in a variety of other scientific disciplines, including systematics, phytogeography, phylogeny, and genome sequencing projects, since their scale and cost depend on this parameter [21–25].

For the estimation of nuclear DNA content, flow cytometry has become the predominant method of choice, as it is fast, accurate, and relatively inexpensive [26]. According to the Plant DNA C-value database [27], nuclear DNA amounts have been estimated for 57 members of Apiaceae using flow cytometry; however, it should be noted that this database does not include all estimates.

Cultivated carrot has a relatively small genome of approximately 473 Mb per haploid genome [28, 29]. In the genus *Daucus*, nuclear DNA content estimates by flow cytometry have been reported for several wild species and subspecies, as well as cultivated carrots, revealing great variation in the 2C DNA amount (0.847–3.019 pg) [30–33].

Pollen of each plant species can be described by a variety of characteristics, including size, shape, aperture number and features, and exine ornamentation; thus, the study of pollen morphology is especially important for plant identification and taxonomic research, and can help to determine the relationships among taxa at various taxonomic levels. Palynological characteristics also play a significant role in fields such as phylogeny, paleobotany, archeology, and criminology [34–37]. Although, according to PalDat [38] (the largest database for palynological data), many members of different genera in the family Apiaceae have been subjected to palynological investigation, a comprehensive study on pollen of the genus *Daucus* is still lacking.

Since the systematics of *Daucus* remains under debate, revisions with the use of additional data are necessary to better understand the relationships within *Daucus* species. Therefore, the aims of this research were (1) to estimate the nuclear DNA content in 13 *Daucus* taxa (14 accessions) and four closely related non-*Daucus* species, frequently used in previous phylogenetic and cytotaxonomic studies of *Daucus* [15, 17, 18, 39], by flow cytometry; (2) to investigate the pollen morphology of these taxa by light and scanning electron microscopy (SEM); (3) to determine their pollen nucleus status; (4) to evaluate the taxonomic value of these cytogenomic and palynological data; and (5) to explore whether any correlations exist between genome size and pollen features.

## Results

### Nuclear DNA content

**Fig. 1:**
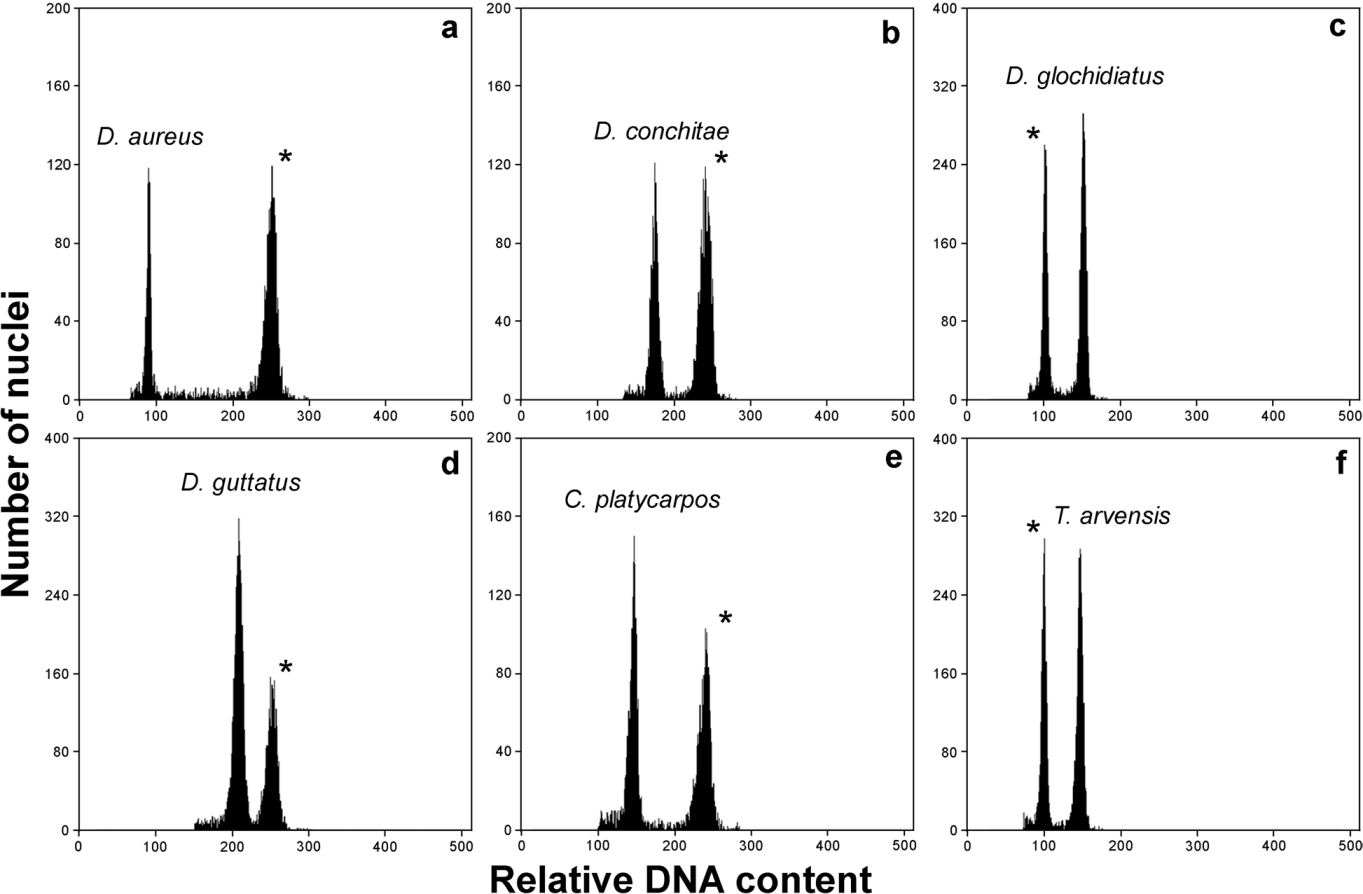
Exemplary histograms of the relative nuclear DNA content of (**a**) *Daucus aureus*, (**b**) *D*. *conchitae*, (**c**) *D*. *glochidiatus*, (**d**) *D*. *guttatus*, (**e**) *Caucalis platycarpos*, and (**f**) *Torilis arvensis*. Asterisks indicate the internal standards: (**a–b**, **d–e**) *Petunia hybrida* ‘P × Pc6’ (2C = 2.85 pg), (**c**, **f**) *Solanum lycopersicum* ‘Stupicke’ (2C = 1.96 pg)

The 2C values for *Daucus* taxa ranged from 0.999 (*D*. *carota* subsp. *sativus* [DH]) to 3.228 pg (*D*. *littoralis*), giving an overall variation of about 3.2-fold (Table 1, Fig. 1). Among the outgroup species, the 2C values varied from 1.775 (*C*. *platycarpos*) to 2.882 pg (*T*. *arvensis*). In both groups, the accessions differed in nuclear DNA content (*p* < 0.001).

**Table 1:** Nuclear DNA content of *Daucus* taxa and outgroup species

Taxon	2*n* ^a^	Ploidy level	Nuclear DNA content			
			N ^b^	2C value (pg, mean ± SE)	2C value range (pg)	1C*x* (pg)
***Daucus*** **I subclade**						
* D*. *aureus*	22	2*x*	21	1.020 ± 0.005 j	0.981–1.059	0.510
* D*. *carota* subsp. *capillifolius*	18	2*x*	20	1.058 ± 0.004 i	1.028–1.089	0.529
* D*. *carota* subsp. *sativus* (DH)	18	2*x*	8	0.999 ± 0.002 j	0.994–1.011	0.500
* D*. *carota* subsp. *sativus* (Dol)	18	2*x*	15	1.003 ± 0.004 j	0.978–1.026	0.501
* D*. *muricatus*	22	2*x*	14	2.126 ± 0.005 d	2.103–2.162	1.063
* D*. *rouyi*	20	2*x*	16	1.129 ± 0.007 h	1.097–1.178	0.565
* D*. *sahariensis*	18	2*x*	10	1.058 ± 0.009 i	1.019–1.129	0.529
* D*. *syrticus*	18	2*x*	4	1.038 ± 0.013 ij	1.010–1.070	0.519
***Daucus*** **II subclade**						
* D*. *conchitae*	22	2*x*	14	2.078 ± 0.004 e	2.037–2.095	1.039
* D*. *glochidiatus*	44	4*x*	16	2.803 ± 0.011 b	2.758–2.902	0.701
*D*. *guttatus*	20	2*x*	14	2.365 ± 0.006 c	2.323–2.410	1.183
* D*. *involucratus*	22	2*x*	11	1.953 ± 0.004 f	1.933–1.976	0.976
* D*. *littoralis*	20	2*x*	19	3.228 ± 0.009 a	3.150–3.318	1.614
* D*. *pusillus*	22	2*x*	22	1.346 ± 0.003 g	1.325–1.378	0.673
**Outgroups**						
* Caucalis platycarpos*	20	2*x*	16	1.775 ± 0.003 d	1.761–1.800	0.888
* Orlaya daucoides*	16	2*x*	23	2.194 ± 0.004 c	2.159–2.244	1.097
* O*. *daucorlaya*	14	2*x*	12	2.625 ± 0.005 b	2.589–2.655	1.313
* Torilis arvensis*	12	2*x*	19	2.882 ± 0.006 a	2.838–2.933	1.441

^a^ The 2*n* chromosome numbers were taken from [39]

^b^ *N*; number of plants

The taxa belonging to the *Daucus* I subclade exhibited lower genome size compared to the taxa from the *Daucus* II subclade, except for *D*. *muricatus* (*Daucus* I), whose genome size was much higher than the other accessions of this subclade and even higher than some species of *Daucus* II subclade (Table 1). The monoploid genome size (1C*x*) of all *Daucus* accessions with 18 chromosomes was similar (about 0.5 pg); however, for the species with higher chromosome number it varied from 0.510 to 1.614 pg and did not relate to *n*. Also, in *Daucus* taxa there was no correlation between the nuclear DNA content and chromosome number (r = 0.521, *p* = 0.06).

Means in columns with the same letter were not significantly different at *p* < 0.001. A one-way ANOVA and Tukey’s HSD test were conducted separately for *Daucus* taxa and the outgroup species.

### Pollen viability and palynological characteristics

The means of pollen viability between *Daucus* taxa differed significantly (*p* < 0.001; Table 2). Despite these differences, the pollen viability rate was relatively high (above 70%), except for *D*. *pusillus*, whose pollen exhibited lower viability. Similarly, pollen of outgroup species expressed high viability after Alexander’s staining, and no significant differences were found between compared accessions.

**Table 2:** Palynological characteristics and pollen viability of *Daucus* taxa and outgroup species

Taxon	Pollen morphology					Pollen viability	
	P ^a^ (µm, mean ± SE)	E ^b^ (µm, mean ± SE)	P/E	Shape class ^c^	Size class ^d^	N ^e^	%, mean ± SE
***Daucus*** **I subclade**							
* D*. *aureus*	40.38 ± 0.08 a	18.14 ± 0.04 c	2.23	Perprolate	Medium	1658	93.6 ± 1.8 a
*D*. *carota* subsp.							
*capillifolius*	27.97 ± 0.08 fg	16.69 ± 0.05 d	1.68	Prolate	Medium	1834	89.6 ± 4.6 ab
*D*. *carota* subsp.							
*sativus* (DH)	26.42 ± 0.06 h	15.46 ± 0.05 f	1.71	Prolate	Medium	2145	75.0 ± 2.2 ab
*D*. *carota* subsp.							
*sativus* (Dol)	26.51 ± 0.07 h	14.31 ± 0.04 h	1.85	Prolate	Medium	2080	91.6 ± 1.1 a
* D*. *muricatus*	38.59 ± 0.07 b	20.21 ± 0.04 a	1.91	Prolate	Medium	1232	95.5 ± 0.3 a
* D*. *rouyi*	33.30 ± 0.04 d	16.23 ± 0.03 e	2.05	Perprolate	Medium	1499	98.6 ± 0.5 a
* D*. *sahariensis*	27.73 ± 0.07 g	16.21 ± 0.06 e	1.71	Prolate	Medium	1542	82.7 ± 8.5 ab
* D*. *syrticus*	27.58 ± 0.08 g	14.74 ± 0.05 g	1.87	Prolate	Medium	306	98.7
***Daucus*** **II subclade**							
* D*. *conchitae*	31.78 ± 0.08 e	16.89 ± 0.05 d	1.88	Prolate	Medium	1676	87.2 ± 8.2 ab
* D*. *glochidiatus*	21.19 ± 0.08 k	15.34 ± 0.06 f	1.38	Prolate	Small	798	93.8 ± 4.3 a
* D*. *guttatus*	28.27 ± 0.10 f	16.75 ± 0.05 d	1.69	Prolate	Medium	1528	73.7 ± 5.5 ab
* D*. *involucratus*	24.86 ± 0.05 i	13.20 ± 0.04 i	1.88	Prolate	Small	1721	96.1 ± 1.6 a
* D*. *littoralis*	37.46 ± 0.10 c	19.26 ± 0.05 b	1.94	Prolate	Medium	1615	96.2 ± 0.8 a
* D*. *pusillus*	21.80 ± 0.08 j	14.87 ± 0.05 g	1.47	Prolate	Small	1098	60.9 ± 13.7 b
**Outgroups**							
* Caucalis platycarpos*	41.90 ± 0.06 c	20.54 ± 0.03 c	2.04	Perprolate	Medium	1889	78.3 ± 5.5 a
* Orlaya daucoides*	42.85 ± 0.10 b	20.89 ± 0.06 b	2.05	Perprolate	Medium	1352	79.9 ± 14.0 a
* O*. *daucorlaya*	49.86 ± 0.15 a	23.54 ± 0.06 a	2.12	Perprolate	Medium	998	96.3 ± 0.9 a
* Torilis arvensis*	26.01 ± 0.05 d	12.06 ± 0.02 d	2.16	Perprolate	Medium	1647	90.7 ± 3.3 a

^a^ *P*, polar axis

^b^ *E*, equatorial diameter

^c^ According to the nomenclature of Erdtman [40]

^d^ According to the nomenclature of Halbritter et al. [36]

^e^ *N*, total number of analyzed pollen grains

The means of the pollen morphometric characteristics (the lengths of the polar and equatorial axes) differed significantly (*p* < 0.001; Table 2) within both *Daucus* taxa and the outgroup species. Following the nomenclature for pollen size by Halbritter et al. [36], which classifies pollen as very small (< 10 µm), small (10–25 µm), medium (26–50 µm), large (51–100 µm), or very large (> 100 µm), our observations revealed that the vast majority of *Daucus* taxa (~ 79%) had medium-sized pollen grains; only three taxa (~ 21%) had small pollen grains. The mean length of the polar axis (P) varied from 21.19 (*D*. *glochidiatus*) to 40.38 µm (*D*. *aureus*). In all outgroup species, the pollen grains were classified as medium in size, of which the pollen of *Orlaya daucorlaya* showed the highest P parameter (49.86 µm). The average equatorial diameter ranged from 13.20 to 20.21 µm in *Daucus* taxa and from 12.06 to 23.54 µm in the outgroup species. Based on the mean ratio of the polar axis to equatorial diameter (P/E), the pollen grains of *Daucus* taxa were classified as prolate (P/E = 1.33–2.00), except for *D*. *aureus* and *D*. *rouyi*, whose pollen were perprolate (P/E > 2.00) in shape, whereas all the outgroup species had perprolate-shaped pollen.

Means in columns with the same letter were not significantly different at *p* < 0.001. A one-way ANOVA and Tukey’s HSD test were conducted separately for *Daucus* taxa and the outgroup species.

**Fig. 2:**
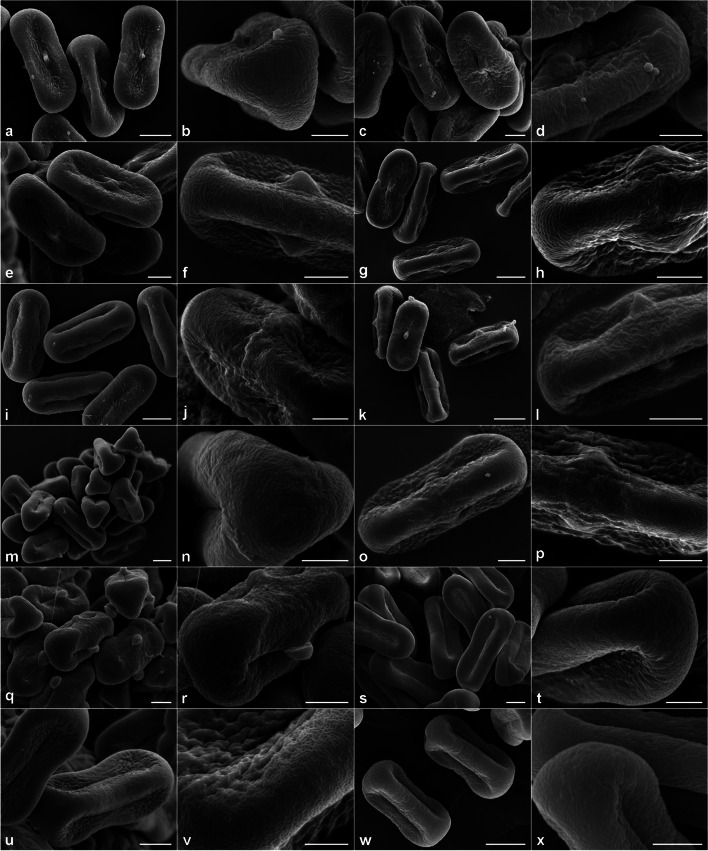
Pollen morphology and exine ornamentation of selected *Daucus* and related taxa by scanning electron microscopy. (**a–b**) *D. conchitae*; (**c–d**) *D. carota* subsp. *capillifolius*; (**e–f**) *D. carota* subsp. *sativus* (DH); (**g–h**) *D. carota* subsp. *sativus* (‘Dolanka’); (**i–j**) *D. guttatus*; (**k–l**) *D. involucratus*; (**m–n**) *D. littoralis*; (**o–p**) *D. rouyi*; (**q–r**) *D. sahariensis*; (**s–t**) *Orlaya daucoides*; (**u–v**) *O. daucorlaya*; (**w–x**) *Torilis arvensis*. Scale bars: 5 µm (**b–f**, **h**, **j**, **l**, **n–r**, **t**, **v**, **x**), 10 µm (**a**, **g**, **i**, **k**, **m**, **s**, **u**, **w**)

The SEM analysis performed on pollen samples of 11 taxa (12 accessions) revealed that the pollen grains were tricolporate with narrow colpori (Fig. 2). Considering the pollen outline in the polar view, the examined taxa had triangular pollen grains (see Fig. 2b, m–n, q). The exine ornamentation was striate (elongated ornamentation elements separated by parallelly arranged grooves) in *D*. *conchitae* (Fig. 2b) and *D*. *rouyi* (Fig. 2p); rugulate (elongated and irregularly arranged ornamentation elements) in *D*. *carota* subsp. *capillifolius* (Fig. 2d) and *D*. *involucratus* (Fig. 2l); perforate in *D*. *guttatus* (Fig. 2j), *O*. *daucoides* (Fig. 2t), and *O*. *daucorlaya* (Fig. 2v), or the ornamentation pattern was mixed, *i*.*e*., striate-perforate in *D*. *carota* subsp. *sativus* (‘Dolanka’) (Fig. 2h), *D*. *littoralis* (Fig. 2n), and *D*. *sahariensis* (Fig. 2r); striate-rugulate in *D*. *carota* subsp. *sativus* (DH) (Fig. 2f); or rugulate-perforate in *T*. *arvensis* (Fig. 2x).

**Fig. 3:**
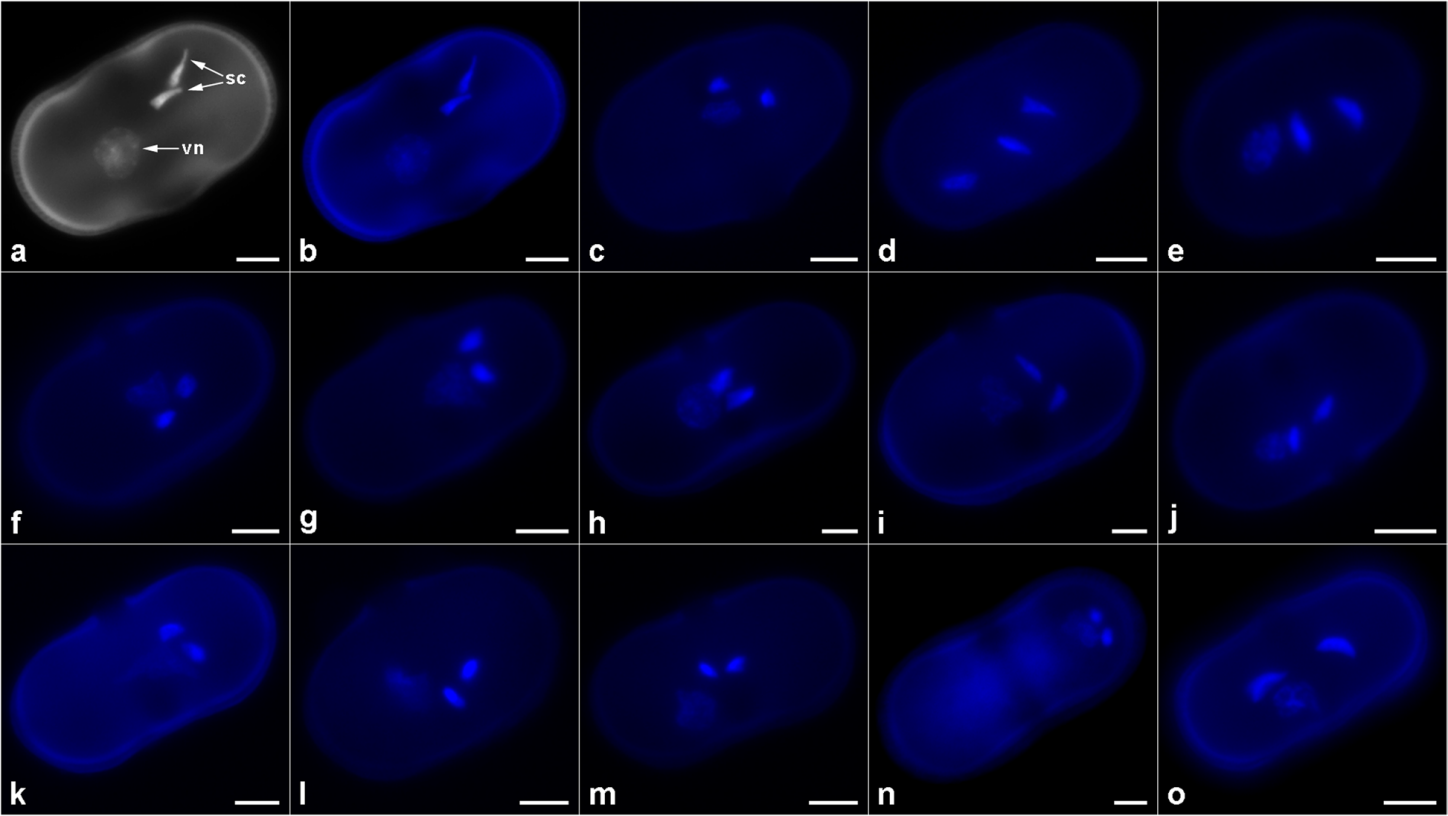
DAPI-stained pollen grains of selected *Daucus* and related taxa. (**a–b**) *D. conchitae*; (**c**) *D. carota* subsp. *capillifolius*; (**d**) *D. carota* subsp. *sativus* (DH); (**e**) *D. glochidiatus*; (**f**) *D. guttatus*; (**g**) *D. involucratus*; (**h**) *D. littoralis*; (**i**) *D. muricatus*; (**j**) *D. pusillus*; (**k**) *D. rouyi*; (**l**) *D. sahariensis*; (**m**) *D. syrticus*; (**n**) *Orlaya daucoides*; (**o**) *Torilis arvensis*. *sc*, sperm cells; *vn*, vegetative nucleus. Scale bar = 5 µm

As evidenced by DAPI staining, at the time of shedding, the pollen of the 14 investigated taxa was three-celled, with a large, weakly stained vegetative nucleus and two smaller, strongly stained sperm cells (Fig. 3). The vegetative nucleus was diffused and more or less round, whereas the sperm cells, depending on the stage of pollen grain development, were round or spindle-shaped and usually located near each other. Although DAPI is a DNA-specific dye, the staining clearly showed the bone-shaped or elliptical outline of the pollen grains, with the apertural areas often visible.

### Interspecific relationships within the genus *Daucus*

**Fig. 4:**
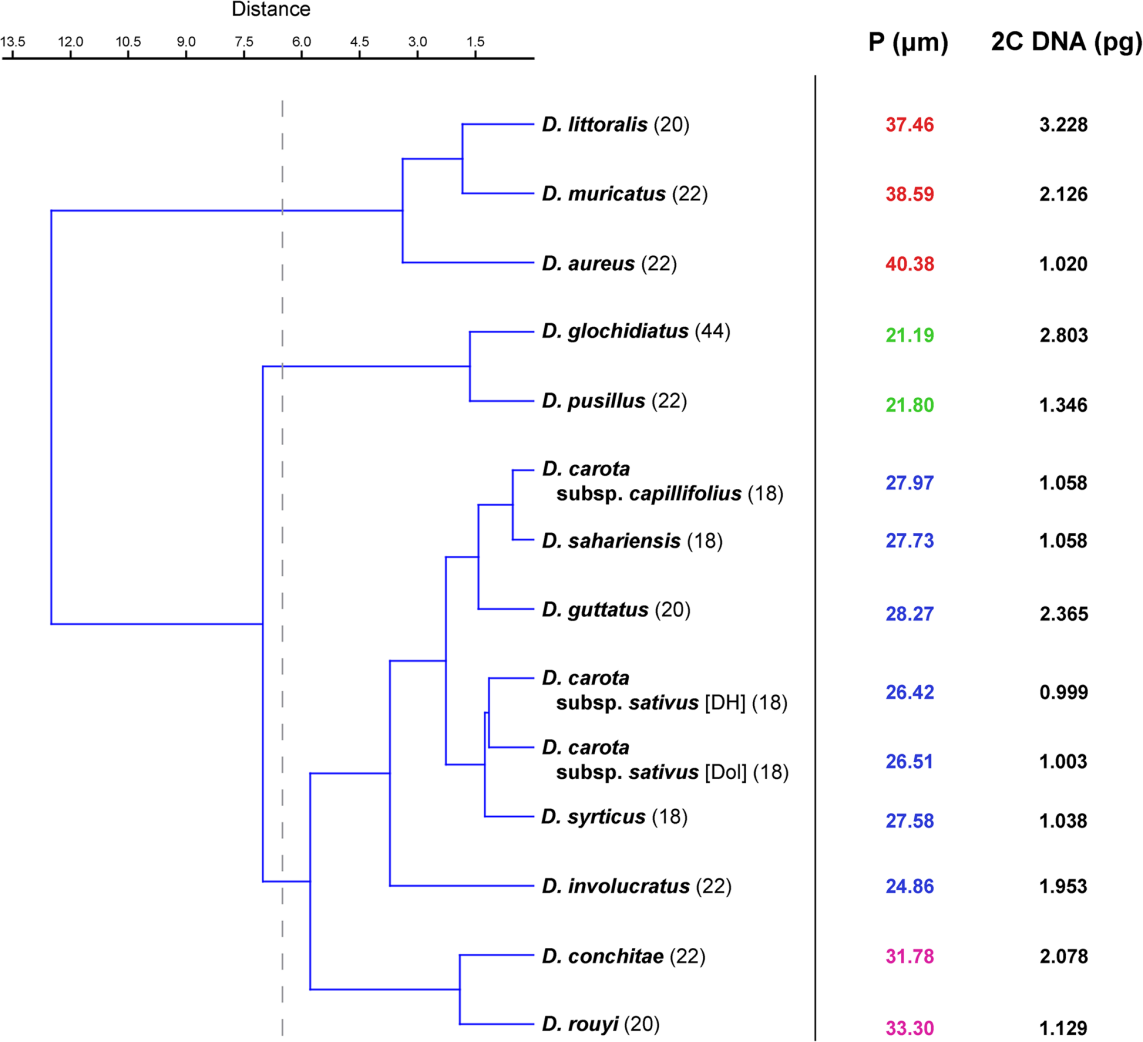
UPGMA similarity dendrogram showing relationships among *Daucus* taxa based on 2C DNA content and pollen characteristics. The 2*n* chromosome numbers are shown in brackets. *P*, the mean size of pollen grains; *Dol*, ‘Dolanka’; the same colors indicate similar values of this parameter

The UPGMA similarity dendrogram, based on three quantitative parameters (2C DNA content, P, E), divided 14 *Daucus* taxa into three major clusters at a Euclidean distance of 6.5, with a cophenetic correlation of 0.86 (Fig. 4). The first cluster contained three taxa with the largest pollen grains but clearly different 2C DNA content (the variation was about 3.2-fold). In the second cluster, two taxa with the smallest pollen grains and an ~ 2.1-fold variation in 2C value were grouped together. The third cluster was subdivided into two subclusters, one of which included the 18-chromosome taxa form *Daucus* I subclade, with very similar pollen size and 2C values, and two taxa from *Daucus* II subclade with an evidently higher 2C value; the second subcluster comprised two taxa with larger pollen grains and a ~ 1.8-fold variation in 2C DNA content.

**Fig. 5:**
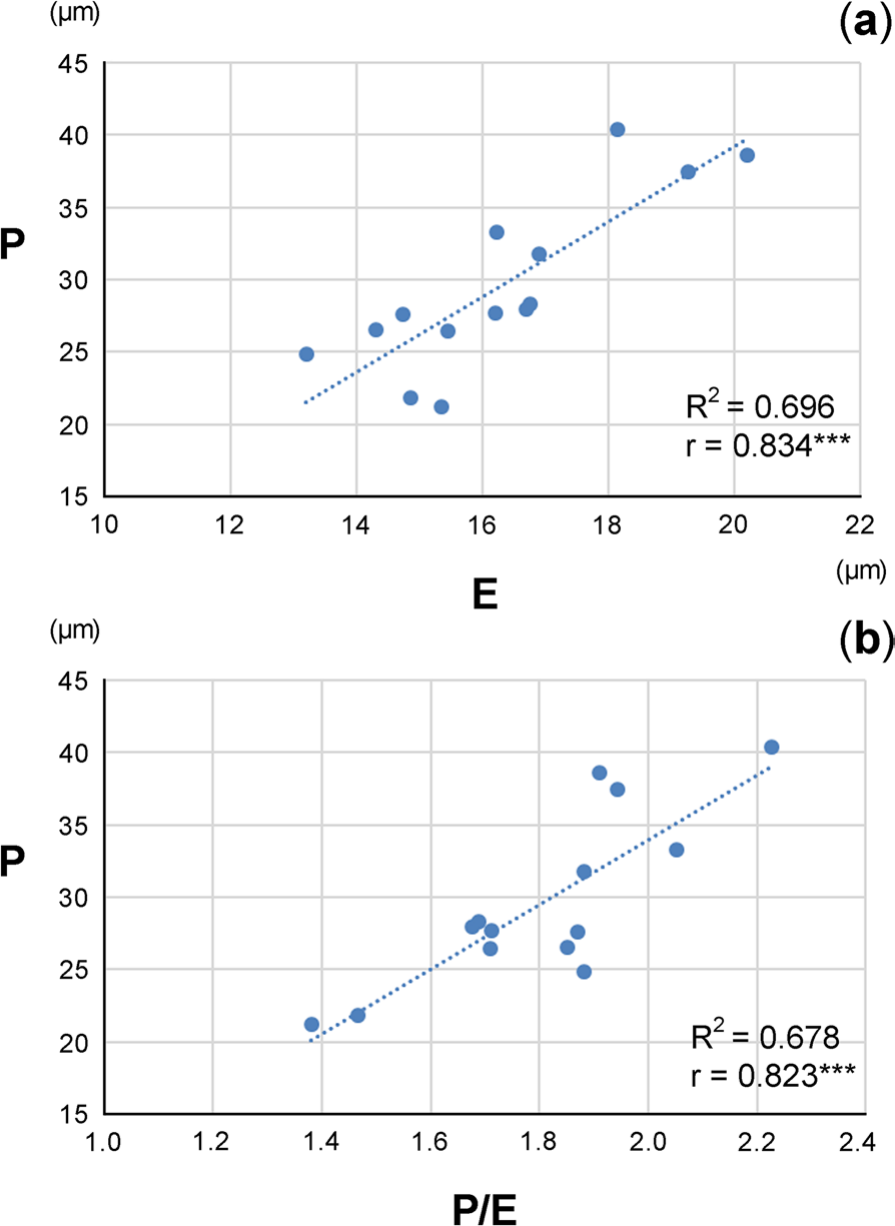
Relationship between (**a**) polar axis (P) and equatorial diameter (E) of pollen grains and (**b**) P and P/E ratio among *Daucus*. Three asterisks (***) indicate statistical significance at *p* < 0.001; *r*, Pearson's correlation coefficient; *R*^*2*^, coefficient of determination

According to the Pearson’s correlation analysis, high positive correlations were found between the length of the polar axis (P) and the length of equatorial diameter (E) of pollen grains (r = 0.834, *p* < 0.001), as well as between P and P/E (r = 0.823, *p* < 0.001) (Fig. 5). No significant correlations were observed when comparing cytogenetic information with palynological data.

## Discussion

In this study, the nuclear DNA content of 13 taxa (14 accessions) of *Daucus* and four closely related species was estimated by flow cytometry, thus expanding the knowledge of genome size variation in the family Apiaceae. Of these, flow cytometric data for 11 taxa are reported here for the first time. Among the studied taxa, almost all had very small genomes (2C ≤ 2.8 pg), except for *D*. *littoralis* and *T*. *arvensis*, whose genome size was categorized as small (2.81–7.00 pg), following the nomenclature of Leitch et al. [41]. It was in agreement with the data included in the Plant DNA C-value database [27], where members of Apiaceae are reported as having mostly very small or small genome sizes; only for a few species genome size exceed 7.00 pg.

To date, the two most extensive cytogenomic studies on the genus *Daucus* have been conducted by Nowicka et al. [32] and Roxo et al. [33]. Nowicka et al. [32] investigated the nuclear DNA content in the collection of diploid members of *Daucus* from different parts of the world, whereas Roxo et al. [33] estimated the 2C values for 16 taxa of the subtribe Daucine from the Macaronesian islands. The results of these works, combined with our findings, revealed an over 3.8-fold variation of the nuclear DNA content within *Daucus*. Some authors suggest that the interspecific variation in DNA content has adaptive significance and correlates with environmental and ecological factors; however, current evidence has been inconclusive so far and does not provide a clear answer as to whether environmental pressure has a relevant impact on plant genome size variation [42–46].

In the present research a low variation (less than 6%) in the 2C DNA content of *D*. *carota* accessions was found. Moreover, all 18-chromosome taxa had similar genome size, which supports the close relationship between these taxa.

Regarding the species for which cytogenomic data have previously been reported, our results are in large part congruent with those obtained by Nowicka et al. [32]. Our estimations for four wild species (*D*. *involucratus*, *D*. *littoralis*, *D*. *muricatus*, and *D*. *pusillus*) were only slightly higher, with a difference of 4–8%, but such discrepancies may be attributed to the different internal standards that were used by the authors (*Brassica napus* L. ‘Bor’ and *D*. *carota* subsp. *sativus* ‘Dolanka’). In contrast, the 2C value obtained here for *D*. *guttatus* conflicted with that of the authors, who found large differences in DNA content for two *D*. *guttatus* accessions. This could result from their taxonomic misclassification because the germplasm of the *D*. *guttatus* complex is especially problematic; thus, misidentifications are frequent [47]. Therefore, in this regard, the results are difficult to compare.

The great differences in the 1C*x* value among wild *Daucus* species suggest that in the course of speciation, large-scale chromosomal rearrangements or the accumulation of non-coding repetitive DNA sequences (particularly retrotransposons) occurred in the genus.

In this study, the pollen morphology of 17 taxa (18 accessions) of the family Apiaceae (13 *Daucus* and 4 non-*Daucus* taxa) was determined, of which 13 taxa (11 *Daucus* and 2 non-*Daucus*) were examined for the first time. In all taxa, tricolporate and prolate–perprolate-shaped pollen were observed, which is a common feature of the pollen grains of Apiaceae [48–52].

Regarding the exine ornamentation observed by SEM, pollen of the examined *Daucus* taxa was striate, rugulate, perforate, or had a mixed ornamentation pattern; thus, this palynological characteristic may be considered useful for species delimitation within *Daucus*. Exine ornamentation plays an important role in plant systematics; it can be useful for distinguishing closely related genera or sometimes species in the same genus [53, 54]. We also compared the data included in PalDat for species of different genera belonging to the family Apiaceae that were also analyzed by SEM (50 species representing 31 genera) and verified that the pollen grains of most of these species are rugulate and perforate (or only rugulate/perforate), suggesting strong homogeneity for this trait in Apiaceae. Nonetheless, outside PalDat, some other types of exine ornamentation in Apiaceae have also been reported in the literature, *e*.*g*., cerebroid, pertectate, and verrucate [50–52, 55].

At the time of shedding, the pollen grains of the studied taxa were three-celled, as revealed by DAPI staining, which is a characteristic feature of pollen in the Apiaceae family [56]. In nature, most flowering plants produce pollen that is arrested at the two-celled stage, containing one vegetative cell and one generative cell, and only around 30% of species shed three-celled pollen (one vegetative cell, two sperm cells) at anthesis [57, 58]. Compared to two-celled pollen, three-celled pollen grains are inherently short-lived [59], and they are also more hydrated [60].

Many examples of correlations between genome size and phenotypic traits at the nuclear, cellular, and tissue levels can be found in the literature. Studies have shown that the amount of DNA is associated with nuclear and cell volume, cell size, cell cycle duration, stomatal cell size, cell density [61–65], seed mass [66], leaf mass per unit area [67], and flowering time [68].

Although the nuclear DNA content did not correlate with the pollen features of *Daucus* taxa, this parameter can be of great use in distinguishing individual taxa within some groups of taxa with a similar pollen size (Fig. 4), *e*.*g*., taxa with small (P < 25 µm) pollen grains (*D*. *glochidiatus*, *D*. *involucratus*, and *D*. *pusillus*) differed in terms of 2C DNA content; the same relation was observed in taxa with P ≈ 30–34 µm (*D*. *conchitae* and *D*. *rouyi*), as well as in those with the largest pollen grains, *i*.*e*., P ≈ 37–41 µm (*D*. *aureus*, *D*. *muricatus*, and *D*. *littoralis*). However, in the group of taxa with pollen grains of P ≈ 26–29 µm, only *D*. *guttatus* can be separated from the others based on 2C DNA content. In the case of whole plant morphology, for example, two morphologically similar species, *D*. *conchitae* (2C = 2.08 pg) and *D*. *guttatus* (2C = 2.37 pg), can be easily distinguished based on their DNA content. On the other hand, some taxa that shared very similar DNA content and pollen size, *e*.*g*., *D*. *carota* subsp. *capillifolius* and carrot, were morphologically distinct.

Wild *Daucus* species may play an essential role in carrot breeding programs, as they could be a valuable potential source of agronomically important genes. Thus, to effectively utilize this germplasm, it is crucial to determine the species boundaries and relationships within *Daucus* [47, 69]. Considering that the correct identification of species is a prerequisite for further use, the application of supplementary methods for this purpose is essential. Therefore, the assessment of relative nuclear DNA content by flow cytometry can be a good choice for simple, rapid, and low-cost screening of genebank accessions during their identification and maintenance, even at seedling stage [24, 70, 71], which could be further combined with palynological measurements to help reliably identify species, as evidenced in this study.

## Conclusions

The present study significantly complements the available information on the nuclear DNA content in the genus *Daucus* and provides comprehensive knowledge of the pollen morphology of its taxa. These results may be of great importance in elucidating the taxonomic relationships among *Daucus* species and can help in the correct identification of gene bank accessions. From a broader view, the findings of this work could also be meaningful for the interpretation of the evolutionary trends in the genus. Nonetheless, to better understand the relationships within *Daucus* in the phylogenetic context, further studies comprising the remaining taxa from the genus, as well as the taxa from different genera that have recently been included in *Daucus*, are needed.

## Methods

### Plant material

A total of 18 accessions representing 17 taxa (species or subspecies) were examined in this study, including 12 accessions of wild *Daucus* taxa, a reference doubled haploid carrot line, one carrot cultivar, and four accessions of closely related non-*Daucus* species (Table 3). Seeds of all wild accessions were provided by the USDA-ARS North Central Regional Plant Introduction Station (Ames, Iowa, USA), whereas carrot seeds were either purchased commercially or obtained from the collections of the Department of Plant Biology and Biotechnology, University of Agriculture in Krakow (Krakow, Poland).

**Table 3:** List of *Daucus* and closely related non-*Daucus* accessions used in this study

Taxon ^a^	Seed source ^b^/Accession no. ^c^	Country of origin
***Daucus*** **I subclade**		
* D*. *aureus*	USDA/PI 319403	Israel
* D*. *carota* subsp. *capillifolius*	USDA/PI 279764	Libya
* D*. *carota* subsp. *sativus* (DH)	RZ/DH1	The Netherlands
* D*. *carota* subsp. *sativus* (Dol)	Commercial/‘Dolanka’	Poland
* D*. *muricatus*	USDA/PI 295863	Spain
* D*. *rouyi*	USDA/PI 674284	Tunisia
* D*. *sahariensis*	USDA/Ames 29096	Tunisia
* D*. *syrticus*	USDA/Ames 29108	Tunisia
***Daucus*** **II subclade**		
* D*. *conchitae*	USDA/Ames 25835	Turkey
* D*. *glochidiatus*	USDA/PI 285038	Australia
* D*. *guttatus*	USDA/PI 652233	Iran
* D*. *involucratus*	USDA/PI 652332	Greece
* D*. *littoralis*	USDA/PI 295857	Israel
* D*. *pusillus*	USDA/PI 349267	Uruguay
**Outgroups**		
* Caucalis platycarpos*	USDA/PI 649446	Germany
* Orlaya daucoides*	USDA/PI 649477	Turkey
* O*. *daucorlaya*	USDA/PI 649478	Greece
* Torilis arvensis*	USDA/PI 649391	Syria

^a^ The taxonomic classification is according to [15, 16]

^b^ *RZ*, Rijk Zwaan vegetable breeding company, Lier, the Netherlands; *USDA*, USDA-ARS North Central Regional Plant Introduction Station (NCRPIS), Ames, Iowa, USA

^c^ *Ames*, Ames numbers are assigned to carrots and other Apiaceae maintained in the NCRPIS; *PI*, USDA Plant Introduction numbers are permanent numbers assigned to germplasm accessions in the National Plant Germplasm System (NPGS); *DH1*, a doubled haploid orange Nantes-type carrot

The seeds were germinated in soil-filled pots and grown in a growth chamber at 18 °C with a long-day photoperiod of 16/8 h (light/dark) for the first few weeks, then transferred to greenhouse conditions (26/14 °C ± 2 °C, day/night temperature; long-day photoperiod) until flowering. In the case of two cultivated carrot accessions, the plants were first vernalized in a cold chamber at 5 °C for three months, then returned to the greenhouse for flowering.

### Flow cytometric measurements of nuclear DNA content

For nuclear DNA content measurements, 8–23 plants per accession, depending on availability, were used. Young leaves were collected from plants grown in a growth chamber and samples for flow cytometric analysis were prepared as previously described [72] using—for nuclei isolation—Galbraith’s buffer [73], supplemented with 1% (w/v) polyvinylpyrrolidone (PVP-10, MW 10,000; Sigma-Aldrich, St. Louis, USA), ribonuclease A (RNase A, 50 µg mL^−1^; Sigma-Aldrich), and propidium iodide (PI, 50 µg mL^−1^; Sigma-Aldrich). *Solanum lycopersicum* L. ‘Stupicke’ (2C = 1.96 pg; [74]) were used as an internal standard for *D*. *glochidiatus*, *D*. *littoralis*, *O*. *daucorlaya*, and *T*. *arvensis*, while for the remaining accessions, *Petunia hybrida* Vilm. ‘P × Pc6’ (2C = 2.85 pg; [75]) was applied. The nuclei suspension was analyzed using a CyFlow SL Green flow cytometer (Partec GmbH, Münster, Germany) equipped with a high-grade solid-state laser (λ_em_ = 532 nm), long-pass filter RG 590 E, DM 560 A, and side and forward scatters. The PI fluorescence was measured in 3000–5000 nuclei per sample. For histogram evaluation, FloMax software (Partec GmbH, Münster, Germany) was applied. The coefficient of variation (CV) of the G_0_/G_1_ peak of sample species ranged between 2.68 and 5.96%. Nuclear DNA content was calculated using the linear relationship between the ratio of the 2C peak positions sample/standard on a histogram of fluorescence intensities.

### Pollen viability, morphology, and nucleus status

Pollen viability was assessed using Alexander’s staining method [76]. Fresh pollen was collected from fully open flowers (from the anthers after dehiscence) of the greenhouse-grown plants onto microscope slides (samples were taken from 1–3 randomly chosen inflorescences per individual, 3–5 plants per accession; except for *D*. *syrticus* and *D*. *glochidiatus*, for which only one and two plants, respectively, were available), then a drop of Alexander’s stain was applied to each slide and covered with a cover glass. The slides were examined under an Axio Imager.M2 microscope (Carl Zeiss, Göttingen, Germany), and the number of viable (dark red cytoplasm with a green wall) and non-viable (entirely green) pollen grains were counted, with a minimum of 300 pollen grains per slide. The pollen viability was expressed as a percentage of viable pollen.

Pollen size was determined using samples of Alexander-stained pollen that had been used for the viability test. The polar axis (P) and equatorial diameter (E) of the pollen grains were measured from microphotographs captured with a Canon PowerShot G10 digital camera (Canon, Tokyo, Japan) attached to the same microscope as above. At least 100 viable pollen grains per plant (3–5 plants per accession; except for *D*. *syrticus* and *D*. *glochidiatus*, for which only one and two plants, respectively, were available) were measured. The terminology for pollen size follows that of Halbritter et al. [36]. The pollen of each accession was classified into a shape class based on the ratio of the polar axis to the equatorial diameter (P/E), according to the nomenclature proposed by Erdtman [40].

For SEM analysis, pollen samples from the fully open flowers of 12 accessions were collected into gelatin capsules and stored in an exsiccator until use. Dry pollen grains were mounted on stubs and sputter-coated with gold using a JFC-1100E ion sputter coater (JEOL, Tokyo, Japan). The palynological characteristics (exine ornamentation and aperture number) were examined under a JSM-5410 scanning electron microscope with a wolfram cathode (JEOL, Tokyo, Japan). The terminology for exine ornamentation follows that of Halbritter et al. [36].

To determine the pollen nucleus status (expressed as the number of pollen nuclei in pollen grains after anther dehiscence), pollen samples of 14 taxa (12 *Daucus* taxa and two outgroup species) were collected in the same way as for the viability test, then mounted in a drop of 4′,6-diamidino-2-phenylindole (DAPI) solution (2.5 µg^−1^ DAPI, 7.7 mM Tris–HCl, 10 mM spermine tetrahydrochloride, 10 mM NaCl, 2.2% hexylene glycol, and 0.25% Triton™ X-100; mixed in a 1:1 ratio with glycerol), and covered with a cover glass. The slides were examined under the same microscope using the fluorescence mode and an appropriate filter set for DAPI (Zeiss filter set 02: λ_ex_ = 365 nm, λ_em_ > 420 nm). The microphotographs were captured using a BV MV camera (Applied Spectral Imaging, Edingen-Neckarhausen, Germany).

### Statistical analyses

For quantitative parameters, means and standard errors of the means were calculated for each accession and subjected to a one-way analysis of variance (ANOVA) followed by Tukey’s honestly significant difference (HSD) test at a significance level of at least *p* = 0.05 using Statistica v. 13.3 (TIBCO Software Inc., USA). For nuclear DNA content estimation and pollen morphology and viability, the mean of measurements/counts for one plant was considered a single replication. Statistical analyses were conducted separately for the *Daucus* taxa and the outgroup species.

To determine the relationships among the *Daucus* taxa, an unweighted pair-group method with arithmetic mean (UPGMA) cluster analysis with Euclidean distance was performed based on nuclear DNA content and pollen morphology (P, E) data using Past v. 3.22 software [77].

To identify relationships among cytogenetic (2C DNA content, somatic chromosome number) and palynological (P, E, P/E) data within the genus *Daucus*, Pearson’s correlation analysis was carried out using Statistical.

## Data Availability

All data generated or analyzed during this study are included in this published article.

## Abbreviations

CWRs: Crop wild relatives
DAPI: 4′,6-Diamidino-2-phenylindole
E: Equatorial diameter
P: Polar axis
SEM: Scanning electron microscopy
UPGMA: Unweighted pair-group method with arithmetic mean

## References

[1] Plunkett GM, Pimenov MG, Reduron JP, Kljuykov EV, van Wyk BE, Ostroumova TA, et al. Apiaceae. In: Kadereit J, Bittrich V, editors. Flowering plants. Eudicots. The families and genera of vascular plants. Cham: Springer; 2018. p. 9–206. 10.1007/978-3-319-93605-5_2.

[2] Plunkett GM, Downie SR (1999). Major lineages within Apiaceae subfamily Apioideae: a comparison of chloroplast restriction site and DNA sequence data. Ann Bot.

[3] Heinonen MI (1990). Carotenoids and provitamin a activity of carrot cultivars (*Daucus carota* L.). J Agric Food Chem.

[4] Sáenz Lain C (1981). Research on *Daucus* L  (Umbelliferae). Anales Jard Bot Madrid.

[5] Rubatzky VE, Quiros CF, Simon PW (1999). Carrots and related vegetable Umbelliferae.

[6] Downie SR, Katz-Downie DS (1996). A molecular phylogeny of Apiaceae subfamily Apioideae: evidence from nuclear ribosomal DNA internal transcribed spacer sequences. Am J Bot.

[7] Downie SR, Katz-Downie DS (1999). Phylogenetic analysis of chloroplast *rps16* intron sequences reveals relationships within the woody southern African Apiaceae subfamily Apioideae. Can J Bot.

[8] Plunkett GM, Soltis DE, Soltis PS (1996). Evolutionary patterns in Apiaceae: inferences based on *matK* sequence data. Syst Bot.

[9] Plunkett GM, Soltis DE, Soltis PS (1996). Higher level relationships of Apiales (Apiaceae and Araliaceae) based on phylogenetic analysis of *rbcL* sequences. Am J Bot.

[10] Vivek BS, Simon PW (1999). Phylogeny and relationships in *Daucus* based on restriction fragment length polymorphisms (RFLPs) of the chloroplast and mitochondrial genomes. Euphytica.

[11] Downie SR, Katz-Downie DS, Watson MF (2000). A phylogeny of the flowering plant family Apiaceae based on chloroplast DNA *rpl16* and *rpoC1* intron sequences: towards a suprageneric classification of subfamily Apioideae. Am J Bot.

[12] Spalik K, Downie SR (2007). Intercontinental disjunctions in *Cryptotaenia* (Apiaceae, Oenantheae): an appraisal using molecular data. J Biogeogr.

[13] Zhou J, Gong X, Downie SR, Peng H (2009). Towards a more robust molecular phylogeny of Chinese Apiaceae subfamily Apioideae: additional evidence from nrDNA ITS and cpDNA intron (*rpl16* and *rps16*) sequences. Mol Phylogenet Evol.

[14] Spooner D, Rojas P, Bonierbale M, Mueller LA, Srivastav M, Senalik D (2013). Molecular phylogeny of *Daucus* (Apiaceae). Syst Bot.

[15] Arbizu C, Ruess H, Senalik D, Simon PW, Spooner DM (2014). Phylogenomics of the carrot genus (*Daucus*, Apiaceae). Am J Bot.

[16] Banasiak Ł, Wojewódzka A, Baczyński J, Reduron JP, Piwczyński M, Kurzyna-Młynik R (2016). Phylogeny of Apiaceae subtribe Daucinae and the taxonomic delineation of its genera. Taxon.

[17] Spooner DM, Ruess H, Iorizzo M, Senalik D, Simon P (2017). Entire plastid phylogeny of the carrot genus (*Daucus*, Apiaceae): concordance with nuclear data and mitochondrial and nuclear DNA insertion to the plastid. Am J Bot.

[18] Spooner DM, Ruess H, Ellison S, Senalik D, Simon P (2020). What is truth: consensus and discordance in next-generation phylogenetic analyses of *Daucus*. J Syst Evol.

[19] Arbizu CI, Ellison SL, Senalik D, Simon PW, Spooner DM (2016). Genotyping-by-sequencing provides the discriminating power to investigate the subspecies of *Daucus carota* (Apiaceae). BMC Evol Biol.

[20] Greilhuber J, Doležel J, Lysák MA, Bennett MD (2005). The origin, evolution and proposed stabilization of the terms ‘genome size’ and ‘C-value’ to describe nuclear DNA contents. Ann Bot.

[21] Doležel J, Greilhuber J (2010). Nuclear genome size: Are we getting closer?. Cytom Part A.

[22] Bennett MD, Leitch IJ (2011). Nuclear DNA amounts in angiosperms: targets, trends and tomorrow. Ann Bot.

[23] Leitch IJ, Leitch AR. Genome size diversity and evolution in land plants. In: Greilhuber J, Dolezel J, Wendel J, editors. Plant genome diversity. Vienna: Springer; 2013. p. 307–22. 10.1007/978-3-7091-1160-4_19.

[24] Yan H, Martin SL, Bekele WA, Latta RG, Diederichsen A, Peng Y (2016). Genome size variation in the genus *Avena*. Genome.

[25] Melichárková A, Španiel S, Marhold K, Hurdu BI, Drescher A, Zozomová-Lihová J (2019). Diversification and independent polyploid origins in the disjunct species *Alyssum repens* from the Southeastern Alps and the Carpathians. Am J Bot.

[26] Doležel J, Greilhuber J, Suda J (2007). Estimation of nuclear DNA content in plants using flow cytometry. Nat Protoc.

[27] Leitch IJ, Johnston E, Pellicer J, Hidalgo O, Bennett MD. Angiosperm DNA C-values database (release 9.0, Apr 2019). 2019. https://cvalues.science.kew.org. Accessed 3 Mar 2022.

[28] Arumuganathan K, Earle ED (1991). Nuclear DNA content of some important plant species. Plant Mol Biol Rep.

[29] Iorizzo M, Ellison S, Senalik D, Zeng P, Satapoomin P, Huang J (2016). A high-quality carrot genome assembly provides new insights into carotenoid accumulation and asterid genome evolution. Nat Genet.

[30] Bai C, Alverson WS, Follansbee A, Waller DM (2012). New reports of nuclear DNA content for 407 vascular plant taxa from the United States. Ann Bot.

[31] Pustahija F, Brown SC, Bogunić F, Bašić N, Muratović E, Ollier S (2013). Small genomes dominate in plants growing on serpentine soils in West Balkans, an exhaustive study of 8 habitats covering 308 taxa. Plant Soil.

[32] Nowicka A, Sliwinska E, Grzebelus D, Baranski R, Simon PW, Nothnagel T (2016). Nuclear DNA content variation within the genus *Daucus* (Apiaceae) determined by flow cytometry. Sci Hortic.

[33] Roxo G, Moura M, Talhinhas P, Costa JC, Silva L, Vasconcelos R (2021). Diversity and cytogenomic characterization of wild carrots in the Macaronesian islands. Plants.

[34] Walsh KAJ, Horrocks M (2008). Palynology: its position in the field of forensic science. J Forensic Sci.

[35] Tuler AC, da Silva T, Carrijo TT, Garbin ML, Mendonça CBF, Peixoto AL (2017). Taxonomic significance of pollen morphology for species delimitation in *Psidium* (Myrtaceae). Plant Syst Evol.

[36] Halbritter H, Ulrich S, Grímsson F, Weber M, Zetter R, Hesse M, et al. Illustrated pollen terminology. 2nd ed. Cham: Springer; 2018. 10.1007/978-3-319-71365-6.

[37] Ullah F, Ahmad M, Zafar M, Parveen B, Ashfaq S, Bahadur S (2022). Pollen morphology and its taxonomic potential in some selected taxa of Caesalpiniaceae observed under light microscopy and scanning electron microscopy. Microsc Res Tech.

[38] PalDat – a palynological database. 2020. https://www.paldat.org. Accessed 10 Jan 2022.

[39] Kadluczka D, Grzebelus E (2021). Using carrot centromeric repeats to study karyotype relationships in the genus *Daucus* (Apiaceae). BMC Genomics.

[40] Erdtman G. Pollen morphology and plant taxonomy. Angiosperms. Leiden: EJ Brill; 1986.

[41] Leitch IJ, Chase MW, Bennett MD (1998). Phylogenetic analysis of DNA C-values provides evidence for a small ancestral genome size in flowering plants. Ann Bot.

[42] Bennett MD (1987). Variation in genomic form in plants and its ecological implications. New Phytol.

[43] Knight CA, Ackerly DD (2002). Variation in nuclear DNA content across environmental gradients: a quantile regression analysis. Ecol lett.

[44] Knight CA, Molinari NA, Petrov DA (2005). The large genome constraint hypothesis: evolution, ecology and phenotype. Ann Bot.

[45] Razafinarivo NJ, Rakotomalala JJ, Brown SC, Bourge M, Hamon S, de Kochko A (2012). Geographical gradients in the genome size variation of wild coffee trees (*Coffea*) native to Africa and Indian Ocean islands. Tree Genet Genomes.

[46] Díez CM, Gaut BS, Meca E, Scheinvar E, Montes-Hernandez S, Eguiarte LE (2013). Genome size variation in wild and cultivated maize along altitudinal gradients. New Phytol.

[47] Arbizu CI, Simon PW, Martínez-Flores F, Ruess H, Crespo MB, Spooner DM (2016). Integrated molecular and morphological studies of the *Daucus guttatus* complex (Apiaceae). Syst Bot.

[48] Perveen A, Qaiser M (2006). Pollen flora of Pakistan – XLVIII. Umbelliferae Pak J Bot.

[49] Güner ED, Duman H, Pinar NM. Pollen morphology of the genus *Seseli* L. (Umbelliferae) in Turkey. Turk J Bot. 2011;35:175–82. 10.3906/bot-0906-70.

[50] Baczyński J, Miłobędzka A, Banasiak Ł. Morphology of pollen in Apiales (Asterids, Eudicots). Phytotaxa. 2021;478(1):1–32. 10.11646/phytotaxa.478.1.1.

[51] Baser B, Sagıroglu M, Dogan G, Duman H (2021). Morphology of pollen in *Ferula* genus (Apiaceae). PhytoKeys.

[52] Birjees M, Ahmad M, Zafar M, Khan AS, Ullah I (2022). Palyno-anatomical characters and their systematic significance in the family Apiaceae from Chitral, eastern Hindu Kush. Pakistan Microsc Res Tech.

[53] Khalik KA, van den Berg RG, van der Maesen LJG, El Hadidi MN (2002). Pollen morphology of some tribes of Brassicaceae from Egypt and its systematic implications. Feddes Repert.

[54] Erden A, Menemen Y (2021). Comparative pollen morphology studies on some species of Brassicaceae in Turkey. Biol Divers Conserv.

[55] Baldemir A, Alan Ş, Şahin AA, Paksoy MY, Pinar NM. Pollen morphology of *Scaligeria* DC. (Apiaceae) in Turkey. Turk J Bot. 2018;42:462–77. 10.3906/bot-1705-43.

[56] Davis GL (1966). Systematic embryology of the angiosperms.

[57] Brewbaker JL (1967). Distribution and phylogenetic significance of binucleate and trinucleate pollen grains in the angiosperms. Am J Bot.

[58] Williams JH, Taylor ML, O'Meara BC (2014). Repeated evolution of tricellular (and bicellular) pollen. Ann Bot.

[59] Lersten NR (2004). Flowering plant embryology: with emphasis on economic species.

[60] Williams JH, Brown CD (2018). Pollen has higher water content when dispersed in a tricellular state than in a bicellular state. Acta Bot Bras.

[61] Jovtchev G, Schubert V, Meister A, Barow M, Schubert I (2006). Nuclear DNA content and nuclear and cell volume are positively correlated in angiosperms. Cytogenet Genome Res.

[62] Beaulieu JM, Leitch IJ, Patel S, Pendharkar A, Knight CA (2008). Genome size is a strong predictor of cell size and stomatal density in angiosperms. New Phytol.

[63] Hodgson JG, Sharafi M, Jalili A, Díaz S, Montserrat-Martí G, Palmer C, et al. Stomatal vs. genome size in angiosperms: the somatic tail wagging the genomic dog? Ann Bot. 2010;105(4):573–84. 10.1093/aob/mcq011. 10.1093/aob/mcq011 PMC2850795 20375204

[64] Hoang PTN, Schubert V, Meister A, Fuchs J, Schubert I (2019). Variation in genome size, cell and nucleus volume, chromosome number and rDNA loci among duckweeds. Sci Rep.

[65] Leitch IJ, Bennett MD. Genome size and its uses: the impact of flow cytometry. In: Doležel J, Greilhuber J, Suda J, editors. Flow cytometry with plant cells. Analysis of genes, chromosomes and genomes. Weinheim: Wiley-VCH; 2007. p. 153–76. 10.1002/9783527610921.ch7.

[66] Beaulieu JM, Moles AT, Leitch IJ, Bennett MD, Dickie JB, Knight CA (2007). Correlated evolution of genome size and seed mass. New Phytol.

[67] Beaulieu JM, Leitch IJ, Knight CA (2007). Genome size evolution in relation to leaf strategy and metabolic rates revisited. Ann Bot.

[68] Comertpay G. Assessment of nuclear DNA contents variation and their relationship with flowering in corn genotypes. Turk J Field Crops. 2019;24(1):39–45. 10.17557/tjfc.562640.

[69] Grzebelus D, Baranski R, Spalik K, Allender C, Simon PW. *Daucus*. In: Kole C, editor. Wild crop relatives: genomic and breeding resources. Vegetables. Berlin: Springer; 2011. p. 91–113. 10.1007/978-3-642-20450-0_7.

[70] Vižintin L, Bohanec B. Measurement of nuclear DNA content of the genus *Trifolium* L. as a measure of genebank accession identity. Genet Resour Crop Evol. 2008;55:1323–34. 10.1007/s10722-008-9331-0.

[71] Rewers M, Jedrzejczyk I (2016). Genetic characterization of *Ocimum* genus using flow cytometry and inter-simple sequence repeat markers. Ind Crops Prod.

[72] Tlałka D, Sliwinska E, Kruk J (2021). *Polystichum setiferum* at the Northestern limit of its distribution range. Acta Soc Bot Pol.

[73] Galbraith DW, Harkins KR, Maddox JM, Ayres NM, Sharma DP, Firoozabady E (1983). Rapid flow cytometric analysis of the cell cycle in intact plant tissues. Science.

[74] Doležel J, Greilhuber J, Suda J. Flow cytometry with plants: an overview. In: Doležel J, Greilhuber J, Suda J, editors. Flow cytometry with plant cells. Analysis of genes, chromosomes and genomes. Weinheim: Wiley-VCH; 2007. p. 41–65. 10.1002/9783527610921.ch3.

[75] Marie D, Brown SC (1993). A cytometric exercise in plant histograms, with 2C values for 70 species. Biol Cell.

[76] Alexander MP (1969). Differential staining of aborted and non-aborted pollen. Stain Technol.

[77] Hammer Ø, Harper DAT, Ryan PD (2001). PAST: paleontological statistics software package for education and data analysis. Palaeontol Electron.

